# Supplementary material for: Sol-Gel Processed TiO2 Nanotube Photoelectrodes for Dye-Sensitized Solar Cells with Enhanced Photovoltaic Performance
Source: Nanomaterials (Basel). 2020 Feb 10;10(2):296. doi: 10.3390/nano10020296 (PMC7075133; doi:10.3390/nano10020296)
Supplement: Supplementary file 1 [file nanomaterials-10-00296-s001.pdf]

Supporting Information

Sol-gel Processed TiO_2 Nanotube Photoelectrodes for Dye-Sensitized Solar Cells with Enhanced Photovoltaic Performance

Nikolay Tsvetkov[1,2,*], Liudmila Larina[1,3], Jeung Ku Kang[2,*] and Oleg Shevaleevskiy[1,*]

[1] Solar Energy Conversion Laboratory, Institute of Biochemical Physics RAS, Moscow 119334, Russia
[2]Graduate School of Energy, Environment, Water, and Sustainability (EEWS), Department of Materials Science and Engineering, Korea Advanced Institute of Science and Technology (KAIST), 291 Daehak-ro, Yuseong-gu, Daejeon, Korea
[3]Department of Chemical Engineering and Applied Chemistry, Chungnam National University, Daejeon 34134, Korea
Email: nickts@kaist.ac.kr, jeungku@kaist.ac.kr; shevale@sky.chph.ras.ru

Section S1. Characterization of Nb-doped NPs and NTs.

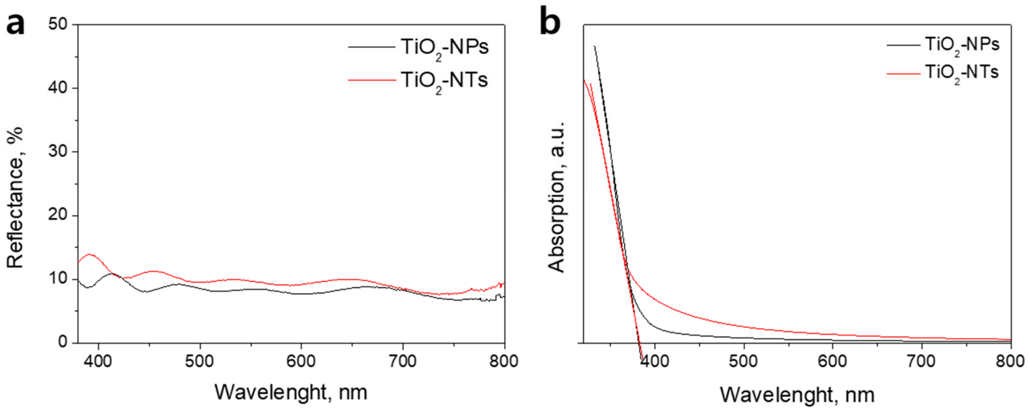

Figure S1. (a) Reflectance of NTs and NPs powders. (b) Absorption spectra of NTs and NPs films.

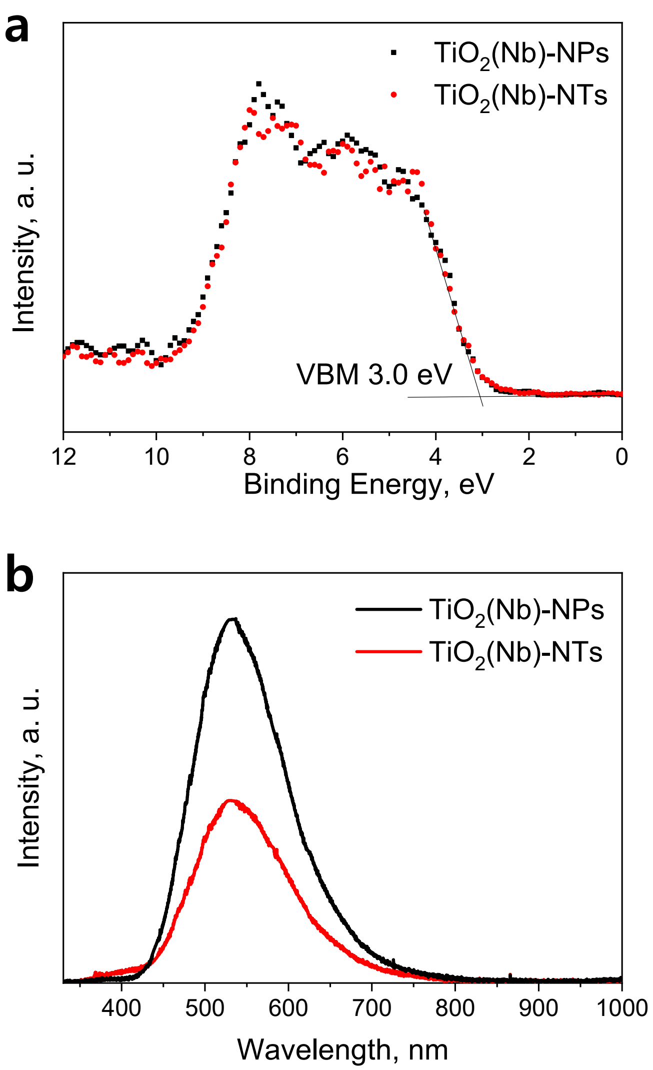

Figure S2. (a) Valence band XPS spectra of TiO_2(Nb)-NPs and TiO_2(Nb)-NTs layers. (b) PL spectra of TiO_2(Nb)-NPs and TiO_2(Nb)-NTs layers taken at 10 K.

Table S1. The ratios of the emission intensity of O p_π to O p_σ orbitals for Nb-doped NPs and NTs.

Sample	O p_π/O p_σ
TiO_2(Nb)-NPs	1.7
TiO_2(Nb)-NTs	2.1

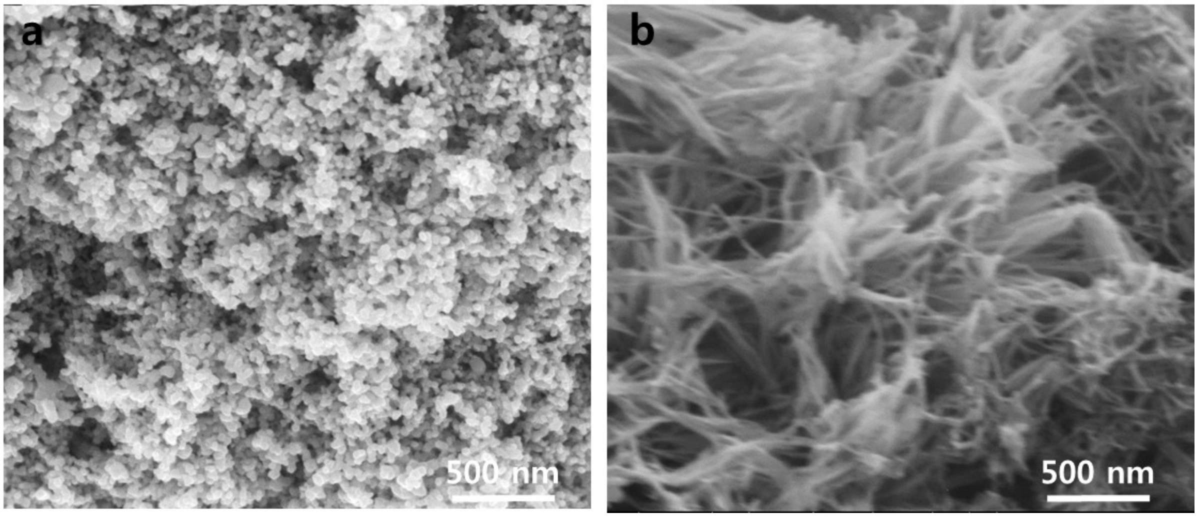

Figure 3. SEM micrographs of (a) TiO_2(Nb)-NPs and (b) TiO_2(Nb)-NTs.
